# Antifouling Strategies of Nanoparticles for Diagnostic and Therapeutic Application: A Systematic Review of the Literature

**DOI:** 10.3390/nano11030780

**Published:** 2021-03-18

**Authors:** Paolo Bevilacqua, Silvia Nuzzo, Enza Torino, Gerolama Condorelli, Marco Salvatore, Anna Maria Grimaldi

**Affiliations:** 1 IRCCS SDN—Via E. Gianturco 113, 80143 Naples, Italy; paolo.bevilacqua@synlab.it (P.B.); direzionescientifica.irccssdn@synlab.it (M.S.); annamaria.grimaldi@synlab.it (A.M.G.); 2 Department of Chemical, Materials Engineering & Industrial Production, University of Naples Federico II, Piazzale Tecchio 80, 80125 Naples, Italy; enza.torino@unina.it; 3 Center for Advanced Biomaterials for Health Care, CABHC, Fondazione Istituto Italiano di Tecnologia IIT@CRIB, Largo Barsanti e Matteucci 53, 80125 Naples, Italy; 4 Department of Molecular Medicine and Medical Biotechnology, “Federico II” University of Naples, Via Tommaso de Amicis 95, 80131 Naples, Italy; gecondor@unina.it; 5 IRCCS Neuromed–Istituto Neurologico Mediterraneo Pozzilli, 18–IT, 86077 Pozzilli, Italy

**Keywords:** biofouling, protein corona, nanoparticle, diagnosis, drug delivery, therapy

## Abstract

Nanoparticles (NPs) are promising platforms for the development of diagnostic and therapeutic tools. One of the main hurdle to their medical application and translation into the clinic is the fact that they accumulate in the spleen and liver due to opsonization and scavenging by the mononuclear phagocyte system. The “protein corona” controls the fate of NPs in vivo and becomes the interface with cells, influencing their physiological response like cellular uptake and targeting efficiency. For these reasons, the surface properties play a pivotal role in fouling and antifouling behavior of particles. Therefore, surface engineering of the nanocarriers is an extremely important issue for the design of useful diagnostic and therapeutic systems. In recent decades, a huge number of studies have proposed and developed different strategies to improve antifouling features and produce NPs as safe and performing as possible. However, it is not always easy to compare the various approaches and understand their advantages and disadvantages in terms of interaction with biological systems. Here, we propose a systematic study of literature with the aim of summarizing current knowledge on promising antifouling coatings to render NPs more biocompatible and performing for diagnostic and therapeutic purposes. Thirty-nine studies from 2009 were included and investigated. Our findings have shown that two main classes of non-fouling materials (i.e., pegylated and zwitterionic) are associated with NPs and their applications are discussed here highlighting pitfalls and challenges to develop biocompatible tools for diagnostic and therapeutic uses. In conclusion, although the complexity of biofouling strategies and the field is still young, the collective data selected in this review indicate that a careful tuning of surface moieties is a pivotal step to lead NPs through their future clinical applications.

## 1. Introduction

Over the past decades, the use of engineered nanoparticles (NPs) has seen a significant increase in the medical field. NPs are classified basing on their physico-chemical characteristics (size, shape, and chemical composition) because it is now accepted that the biological and toxicological effects are strongly correlated with their physical properties [1]. Many studies have already shown that tissue distribution and therapeutic activity are size and charge surface dependent. NPs are extremely versatile vectors that, thanks to their small size (10–100 nm) [2], can cross biological barriers and are able to penetrate organs, tissues, and cells, with this being a reason that they are promising tools for therapeutic and diagnostic purposes [3,4].

**Figure 1:**
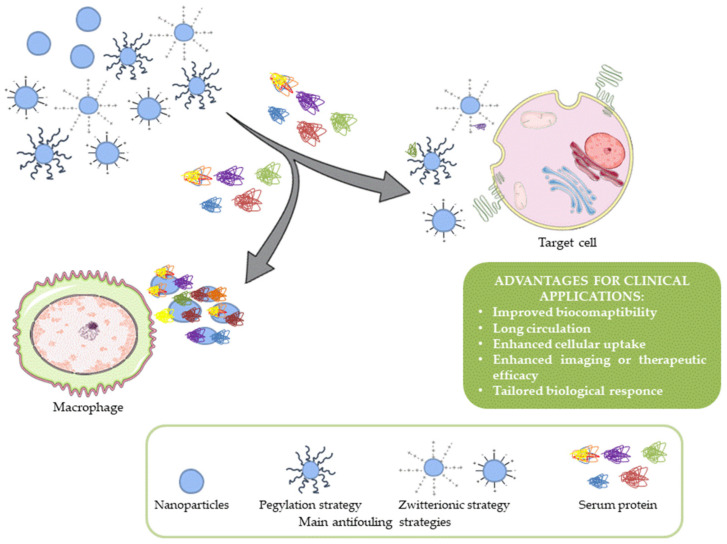
Nanoparticles fate: Scheme of current understanding antifouling mechanisms.

Even if NPs could improve the efficiency of therapeutic and diagnostic agents protecting them from degradation and/or increasing their solubility, only a few NPs are available on market [5]. Nowadays, the clinical application of NPs is still limited due to incomplete knowledge of the interaction with macromolecules present in organic fluids, that absorbing on the surface, determine the “Protein Corona” (PC) formation [6]. The PC, composed of a complex of biomolecules as proteins, sugars, nucleic acids, and lipids, influences NPs performance in vivo, affecting their biodistribution, safety, and toxicological factors [7]. From the first study in 2007, Dawson, Linse, and co-workers for the first time introduced the concept of PC as major obstacle to the application of NPs in vivo [8]. The mechanism of absorption of proteins and other molecules on NPs surfaces is named biofouling, and it is a dynamic process finely regulated by the surrounding microenvironment, as shown in Figure 1. Although some studies demonstrate that PC could reduce the unspecific uptake from cells or increase the stability in vivo of NPs [9], in several studies, the PC formation is considered a disadvantage because by reducing the circulation times of NPs in bloodstream, it impairs their therapeutic or diagnostic activity [10]. In this scenario, the biofouling process has a pivotal role in clinical practice because it not only reduces the efficacy of treatment, but it produces hemolysis, leading to implant rejections [11] or infections [12]. The surface characteristics of NPs (charge, hydrophobicity, or coating) determine the affinity coefficient (kD) for each component of PC, with this being a reason that it is important to develop novel coatings able to prevent biofouling of NPs and consequently improve their targeting and drug delivery [13,14]. This systematic review provides the current state of the art on the design of antifouling coatings of NP surface.

## 2. Materials and Methods

The systematic review was performed to establish if the antifouling strategies could improve the therapeutic and diagnostic value of NPs and prevent side effects. This study did not require ethical approval because the data analysis was carried out based on previously published data.

### 2.1. Literature Search and Study Selection

Three scientific electronic databases (PubMed, MEDLINE, and Google Scholar) were used to conduct a systematic literature search. Only studies published since 2009 were selected. The key terms used for the search strategy are listed in Supplementary Materials (S1. Key Terms Used in Literature Search). Briefly, the search included antifouling strategies developed for clinical applications (diagnosis and therapy) and assessed in vitro and or in vivo experiments. Two authors independently reviewed the articles for eligibility from titles and abstracts. The full article was checked when it met the inclusion criteria but the information was not clear only in the title and abstract. Finally, we included only manuscripts dealing with the utility of antifouling strategies for clinical applications. The exclusion criteria involved non-English papers, reviews, metadata, single case reports, letters to the editor, methodological studies, and papers regarding the development of sensors. The entire selection flow and results of the literature research were checked by a third researcher.

### 2.2. Data Extraction and Collection

The selected articles were analyzed by two reviewers, and the principal data were extracted and collected in a pre-designed sheet. We summarized extracted data including main study characteristics (first author name, year of publication, nanostructure, antifouling moiety, in vivo and/or in vitro model, application) in Table 1 and Table 2.

### 2.3. Planning and Conducting the Review

We conducted this Systematic Review in accordance with the Preferred Reporting Items for Systematic Reviews and Meta-Analyses (PRISMA) statement (for more details see PRISMA Check list in Supplementary Materials). The articles were classified according their main purpose, i.e., whether they reported antifouling strategies applied for diagnosis or therapy.

## 3. Results

### 3.1. Study Selection

**Figure 2:**
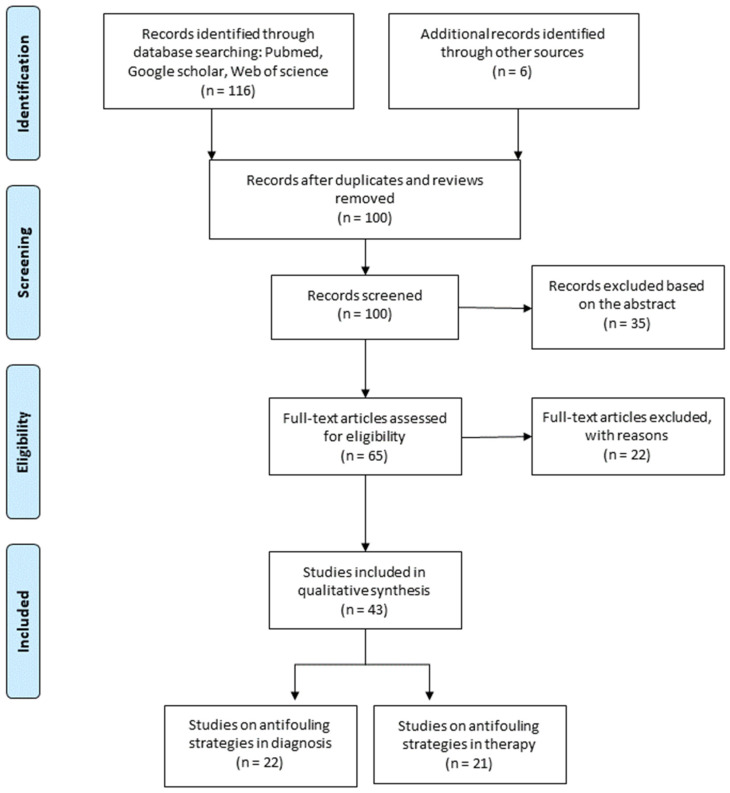
PRISMA flow diagram. From: Moher D, Liberati A, Tetzlaff J, Altman DG, The PRISMA Group (2009). Preferred Reporting Items for Systematic Reviews and Meta-Analyses: The PRISMA Statement. PLoS Med 6(7): e1000097, doi:10.1371/journal.pmed1000097. For more information, visit: www.prisma-statement.org.

A flowchart of the publication search and the detailed selection process of the articles is reported in Figure 2. A total of 122 potential eligible records related to antifouling coatings of NPs surface for diagnostic and therapeutic purposes were retrieved from public databases and additional sources, such as relevant studies identified by references of other scientific papers. Then, 22 duplicates were deleted, and of the remaining 100 records, 32 were excluded after screening the abstract because they were irrelevant. Then, the remaining eligible articles (65) were all downloaded and read, and 22 of them were excluded due to the paucity of sufficient information. The final 43 eligible articles were grouped according to two fields of study: (1) Antifouling strategies for diagnostic purpose, and (2) antifouling strategies for therapeutic purpose. Here, we identified two main antifouling macro areas: PEGylation and zwitterionic polymers. PEGylation is a useful strategy to increase blood circulation time for imaging and drug delivery benefiting passive targeting promoted via the enhanced permeability and retention (EPR) effect. On the other hand, zwitterionic polymers are considered as the next generation of antifouling materials because they are capable of forming hydration shells through electrostatic interactions (much more resistant than hydrogen bonds), and they can be easily functionalized and designed. Surfaces modified with zwitterion ligands are more biocompatible, bioinert, and do not induce any production of antibodies as PEG. Furthermore, some zwitterions are stimuli-responsive, offering a remarkable strategy to improve drug delivery and control time-release preventing cargo premature leakage.

### 3.2. Antifouling Strategies for Diagnostic Purpose

In this paragraph, we discuss 22 articles exploiting the antifouling properties of NP systems developed for imaging and diagnostic purposes. All selected characteristics are summarized in Table 1. Depending on the antifouling moieties used, we grouped these studies into three subgroups: (i) PEGylation strategy, (ii) zwitterionic strategy, and (iii) other strategies.

#### 3.2.1. PEGylation Strategy

Among the 22 selected studies assessing the antifouling properties of NP for imaging and diagnostic purposes, seven of them pursued this goal exploiting PEGylation strategy. The first example of NPs coated with Polyethylene glycol (PEG) for diagnostic imaging dates back to 2009 and consists of cross-linked superparamagnetic iron oxide NPs (TCL-SPION). Cho et al. [15] assessed the toxicity and kinetic profile of antifouling polymer coated Cy5.5-conjugated to SPION in BALB/c mice. These NPs, prepared as diagnostic probes for optical imaging, induced slight pulmonary inflammation and no toxicity at 1.8 mg/kg concentration. Moreover, the kinetic study revealed that NPs were rapidly eliminated from the lung mice, and their fluorescence intensity decreased by about 4 times at 24 h after treatment. Two years later, Oh et al. [16] functionalized PEGylated tantalum oxide (TaOx) NPs with a fluorescent dye (Rhodamine-B-isothiocyanate, RITC) for bimodal imaging. Authors verified that PEGylation endowed the NPs with biocompatibility and antifouling activity: Cell viability was not impaired up to a concentration of 2.4 mg of Ta/mL at 24 h, and cellular uptake in murine macrophage cells (RAW264.7) resulted in being dose-dependent. Moreover, the authors demonstrate that, after the injection of 840 mg/kg of PEG-RITC-TaOx in in vivo rat model, the PEGylation ensured NPs long-circulating and their tracking demonstrated that the vessels were preferentially enhanced for over 3 h after. Moreover, the NPs after 24 h mainly accumulated in the liver and spleen and no adverse effector histological changes were found on tissue. Besides, bimodal image-guided surgery performed by using these nanosized as bimodal contrast agents resulted advantageously for the resection of lymph nodes. Another example of PEGylated NPs for multimodal imaging was provided by Liu et al. [17]. Here, authors synthesized PEGylated hybrid lutetium oxide nanoprobes (PEG–UCNPs) and assessed their colloidal stability in various solutions including fetal bovine serum (FBS), phosphate-buffered saline (PBS), and Dulbecco’s modified Eagle medium (DMEM) cell media. Cell viability was assessed after 48 h of co-incubation with PEG–UCNPs (200 µg/mL) and the cells spread and proliferated equally to the control group, without exhibiting cytotoxic profile. Moreover, the authors demonstrated high blood compatibility in in vivo models and biodistribution studies showed that PEG–UCNPs accumulated in the spleen and liver. Moreover, the marked decreasing signal of Lu^3+^ contents in all organs after intravenous administration within the first month indicated a stepwise clearance route mainly via renal and fecal excretions in a murine model.

Jeong et al. [18] exploited antifouling properties of PEG modifying it and synthesizing novel polymeric poly(oxyethylene galactaramide)s (PEGAs), used for producing self-assembled NPs useful for diagnostic imaging. First, the authors assessed their antifouling properties in vitro experiments, where NPs appeared not internalized into cells, but only confined in the extracellular compartment. Then, antifouling properties of the self-assembled PEGA NPs were assessed also in vivo by near-infrared fluorescence imaging (NIRF), where they were able to cross biological barriers, circulate in the bloodstream, and accumulate into tumor tissue. A similar strategy was assessed by Li et al. [19] that coated magnetic iron oxide NPs with a new copolymer made of blocks of PEG and allyl glycidyl ether (PEG-b-AGE) for (IONPs). These NPs exhibited antifouling properties with reduced PC formation and without aspecific macrophage uptake. Furthermore, when functionalized with targeting ligands such as Transferrin (Tf) and arginine-glycine-aspartic acid peptide (RGD) their antifouling properties were preserved and targeting capabilities, resulting in being significantly improved. Moreover, the reduced specific macrophage uptake positively affected relaxometry results, improving the specificity of Magnetic Resonance Imaging (MRI) contrast change. Tu et al. [20] used PEG together with bovine serum albumin (BSA) to coat monodisperse Silicon Quantum Dot NPs (SiQD-NPs) of diameter about 130 nm. The terminal functional groups of the antifouling layer were then used for conjugating anti-HER2 antibody; indeed, these NPs were proposed as a diagnostic tool for immunostaining on hepatic cancer cells (SKOV3). SiQD-NPs revealed high biocompatibility at in vitro cell viability assay, showed minimal cytotoxicity after 48 h of treatment (IC_50_ =1600 μg/mL) and were able to produce specific immunofluorescence images comparable to FITC. Recently, Garcia et al. [21] realized pegylated metal-phenolic nanoparticles (MPS-PEG) with a polymeric core that combined radioactive metals such as ^111^In or ^64^Cu. Furthermore, PEG was used as an anchoring platform to attach folic acid (FA) to facilitate in vivo targeting and multifunctionality. PEG coating affected particle biodegradability, allowing controlled metal release in biological milieu over a pH range and prevented cell toxicity at metal concentration up to 200 µM. Moreover, these particles provided a strong PET signal in lung when assessed in mice bearing CT26 tumors.

#### 3.2.2. Zwitterionic Strategy

We found 11 studies that used several kinds of zwitterionic strategies for coating NPs and proposed them as useful imaging contrast agents, the first one reported here regarding the use of Cysteine (Cys). Li et al. [22] used L-cysteine (L-cys) to develop degradable zwitterionic polymers, named zPPE, for gold NPs (AuNPs), and assessed this nanoplatform for drug delivery, molecular imaging, and photothermal therapy. The coated NPs showed negligible immunotoxicity, in fact mouse-derived macrophages (RAW 264.70) incubated with AuNP formulations showed levels of 23 cytokines comparable with untreated cells. Zwitterionic properties of Cys were exploited also to synthesize ultrasmall Gadolinium oxide NP (Gd_2_O_3_-PEG-Cys-NPs) for MRI purposes [23]. Although both Gd_2_O_3_-PEG-Cys-NPs and Gd_2_O_3_-PEG exhibited overlapping cytocompatible and hemocompatible profiles in the concentration range of 5–25 μg/mL, the former exhibited improved antifouling behavior compared with the starting material as demonstrated by cellular uptake in vitro, and by improved pharmacokinetic properties in vivo (half-life of 6.2 h). Moreover, Gd_2_O_3_-PEG-Cys-NPs showed better efficiency as MRI contrast agents. The antifouling property of L-cys was also exploited for modifying the surface of Manganese Oxide(Mn_3_O_4_) NPs [24]. L-cys (4 μg/μL) decreased cellular uptake and prolonged blood circulation time until 28.4 h. The same group prepared Cys-coated ultrasmall Fe_3_O_4_ NPs for MRI applications [25]. Fe_3_O_4_-PEG-Cys NPs displayed colloidal stability, negligible cytotoxicity, and hemocompatibility (studied concentration range 0–100 µg/mL) and better antifouling property.

A few years later, the same authors [26] synthesized antifouling L-lysine (L-lys) zwitterion-functionalized Mn_3_O_4_ NPs modified with folic acid (FA) for targeted tumor MRI. The surface decoration of L-lys endowed the NPs with good antifouling properties as suggested by the fact that at the same Mn concentration the zwitterionic Mn_3_O_4_ displayed a much lower bovine serum albumin (BSA) adsorption than those without L-lys modification. Further L-lys modified NPs showed a larger relaxivity in vitro, much higher than the Mn_3_O_4_ NPs described in their previous work [24]. L-lys zwitterion-FA functionalized Mn_3_O_4_ NPs significantly, delivered to the target site after intravenous injection (5 μg/μL), in xenografted tumor mouse models, following improved targeted tumor MRI. Without L-lys decoration, the NPs were largely cleared by the reticuloendothelial system (RES).

Another kind of zwitterionic strategy is offered by using carboxybetaine (CBAA). Xiong et al. [27] used antifouling zwitterion CBAA to modify the surface of dendrimer-entrapped gold NPs (Au DENPs) to apply for CT imaging. Au DENPs displayed better antifouling properties than PEG-modified counterparts. CBAA modification onto NP surface rendered NPs with a layer of water, which gave antifouling properties such as reduced protein resistance, higher cellular uptake, and longer half-delay time, thus they could passively accumulate in the tumor site. Moreover, a higher CBAA modification degree on NP surface significantly improved CT contrast enhancement for lymph node and tumor imaging. Another study entrapped Au NPs into poly(amidoamine) (PAMAM) dendrimers and decorated them with carboxybetaine acrylamide (CBAA), 1,3-propane sultone (1,3-PS), and 2-methacryloyloxyethyl phosphorylcholine (MPC), for developing enhanced dual-mode imaging [28]. Among all, zwitterionic Au DEN modified with 1,3-propane sultone (1,3-PS) exhibited the best antifouling property with better performance of protein resistance, aspecific cellular uptake, and significantly longer half-decay time compared with CBAA and MPS modification. These nanoplatforms, further modified with RGD peptide, displayed a high relaxivity, cytocompatibility, targeting specificity to cancer cells, and allowed dual-mode imaging of metastatic lung cancer in vivo model. 1,3-PS was also used to provide antifouling property to multifunctional poly(cyclotriphosphazene-co-polyethylenimine) nanospheres (PNSs), labelled with radionuclide ^131^I, and realized for single-photon emission computed tomography (SPECT) imaging-guided radiotherapy of tumors [29]. Multifunctional PNSs with an average diameter of 184 nm exhibited cell viability above 81.7 ± 2.21% in the assessed range of concentration (0–100 µM/mL), colloidal stability, and high ^131^I labelling efficiency (76.05 ± 3.75%). Moreover, the developed nanospheres showed long retention (until 24 h after administration) in the tumor region, useful for their uses for SPECT imaging-guided tumor radiotherapy.

Recently, biocompatible magnetic NPs were developed throughout the functionalization with zwitterionic dopamine sulfonate (FeOx/ZDS NPs) by Ferretti et al. [30]. Zwitterionic NPs demonstrated colloidally stability, good protein resistance, and hemocompatibility. Furthermore, coated NPs did not trigger innate/inflammatory reactions and were scarcely engulfed by phagocytic (BV2) cells. Furthermore, in vivo MRI studies revealed that these NPs (1 or 4 mg Fe/kg) had rapid renal clearance (about 1 h) while some others resided in the liver and spleen for an extended time.

Tasso and colleagues [31] capped quantum dot (QD) NPs with the zwitterion/vinylimidazole block copolymer exploiting amine functionalities for the bioconjugation of IgG antibodies. The obtained nanoconjugates exhibited intracellular stability and considerable efficacy at specifically targeting the transmembrane cannabinoid receptor (CB1R), enabling the multicolor fluorescence imaging on HEK293 and neurons until 48 h.

An alternative zwitterionic strategy was proposed by Wang et al. [32] that exploited surfactant properties with abundant carboxylic groups of BSA as stabilizer and antifouling. Hydrophobic SPIONs capped by BSA exhibited colloidal stability and high relaxivity. Moreover, a monoclonal antibody (mAb) was linked to the BSA equipped SPIONs of high specificity in recognition of Plectin-1, a specific biomarker for pancreatic cancer cells.

#### 3.2.3. Other Strategies

Bifunctional dendrons represent a promising option for combining different functionalizations with the need for biocompatibility and stability. Lamanna et al. [33] grafted dendrons on the surface of iron oxide NPs by using a phosphonate group as a coupling agent to develop new contrast agents. The presence of either neutral and carboxylic groups on the surface favored the stability in iso-osmolar media and at physiological pH. No evident adverse effects were observed after in vivo administration. Although no RES uptake was detected, fast hepatobiliary elimination and low urinary excretion were observed. These dendronized NPs determined a significant MRI signal enhancement in vivo, compared with polymer-coated iron oxides. More recently, Cotin and colleagues [34] also developed NPs with different morphology and functionalized them with dendrons. After 24h, independently from the shape, NPs showed no sign of cytotoxicity up to a concentration of 8 mM. However, with higher doses (at 16 mm), cell mortality was detected, and it resulted in being shape-related for NPs with cubic or spheric morphology. The dendron coating provided good colloidal stability in vitro and prevented NPs from non-specific internalization. Moreover, biodistribution studies in CD-1 mouse model showed the suitability of the four dendronized NPs as contrast agents. The same NPs, when assessed for magnetic hyperthermia, showed limited effect due to low NPs internalization.

Karmali et al. [35] synthesized cross-linked dextran-coated superparamagnetic iron oxide NPs (SPIO). Authors showed that hydrogelation of magnetic nanoworms left the shape, size, and charge unchanged, but decreased their clearance in vivo (1−4 mg Fe/kg body weight), preventing the recognition by Kupffer macrophages in the liver. Moreover, hydrogelation affected the number of hydroxylic groups on the NPs surface, reducing the binding of the anti-dextran antibody, but without impairing the absorption of cationic proteins. In this way, the binding of kininogen, histidine-rich glycoprotein, and protamine sulfate to the anionic NPs surface remained unvaried. Subsequently, the same group developed antifouling PEO-b-PγMPS-coated magnetic nanocrystals for targeting and imaging of breast cancer and assessed their retention time in vivo [36]. NPs were functionalized with the anti-HER2 monoclonal antibody (Herceptin) or single chain antibody fragment (ScFv) against epidermal growth factor receptor (ScFvEGFR). PEO-b-PγMPS diblock copolymer-coated IONPs showed reduced nonspecific uptake by RAW264.7 mouse macrophages and exhibited “stealth” properties in vivo, as proved by a long blood circulation time (serum half-life 11.6) and low accumulation in liver and spleen. Moreover, the administration of ScFvEGFR-IONPs enabled the active targeting of breast cancer cells with targeted imaging.

### 3.3. Antifouling Strategies for Therapeutic Purpose

In this paragraph, we discuss 21 articles that exploited the antifouling properties of NP systems developed as drug delivery systems. The main characteristics of the selected studies are summarized in Table 2. Depending on antifouling moieties used, we grouped these studies into three subgroups: (i) PEGylation strategy; (ii) zwitterionic strategy, and (iii) other approaches.

#### 3.3.1. PEGylation Strategy

Among the 21 selected studies assessing the antifouling properties of NP for therapeutic purpose, only 3of them pursued this goal exploiting PEGylation strategy. Lipidic PEGylated NPs were functionalized with FA in ovarian cancers to improve the active targeting of capsaicin (CAP) [37]. After injection of 10 mg/kg of CAP in rat, the authors demonstrated that the antifouling property of PEG protected lipidic NPs from protein interaction increasing consequently the blood circulation time of the drug. On the other hand, the active targeting allowed reaching specifically to cancer cells with remarkably higher uptake and anticancer effect compared with non-targeted nanosystems. Park et al. [38] designed amphiphilic poly(ethyleneimine) (aPEI) NPs and modified their surface with PEG for multi-cargo delivery. The highly PEGylated NPs (aPEI-25 NPs), tested both in vitro and in vivo, showed (i) good cargo loading capacity and better tumor-delivering capability, (ii) high biocompatibility, and (iii) minimal cytotoxicity at 0.32 mm concentration. Another example of NPs functionalized with PEG was provided by Yao et al. [39]. The authors developed photoresponsive lipid-polymer hybrid NPs for a controlled release of Doxorubicin (DOX). These hybrid NPs were made of three distinct functional components important to (i) encapsulate DOX (poly(D,L-lactide-co-glycolide) (PLGA)), prevent drug leakage (Soybean lecithin monolayer), and enhance NP stability (photoresponsive polymeric shell). The latter shell could be detached by irradiation to decrease the stability of the NPs and to trigger drug release, as demonstrated by increased antitumor efficiency in in vivo HepG2 tumor bearing nude Balb/c mice (3 mg mL−1 DOX loaded LPHNP).

#### 3.3.2. Zwitterionic Strategy

We found 15 studies that exploited zwitterionic moieties for implementing antifouling NPs and demonstrating their effective role as nanocarriers for therapeutic purposes.

Elsabahy et al. [40] synthesized polyphosphoester (PPE)-based micelles to deliver therapeutics and studied the protein adsorption effect and toxicity profiles mediated by shell crosslinking and surface charges. PPE-based micelles, compared to several commercially available vehicles, showed lower cytotoxicity, and their degradation products were also not cytotoxic up to a concentration of 3000 μg/mL in vitro. Thanks to the absence of secretions of any of the 23 measured cytokines in RAW264.7 mouse macrophages, the authors demonstrated that PPE-based micelles caused no immunotoxicity.

Huang [41] and colleagues compared the antifouling properties of conventional coating (11-mercaptoundecyl)tri(ethylene glycol) (OEG-thiol) to hollow gold-silver nanoshells functionalized with cysteine betaine (Cys-b) for hyperthermia applications. Both coatings (Cys-b and OEG-thiol) exhibited comparable antifouling resistance with gram-negative and gram-positive bacteria, NIH-3T3 fibroblasts, and BSA. However, Cys-b nanoshells-modified exhibited great colloidal stability and photothermal properties also at high ionic strength and temperature. Besides, adding anti-HER2 antibodies functionalization to the nanoshell surface, the authors performed aspecific and effective tumor ablation through hyperthermia treatment. Another example of zwitterionic nanocarrier for hyperthermia treatment was provided by Zheng et al. [42]. Authors designed injectable thermosensitive nanogels that combined the effect of photothermal therapy with local chemotherapy for tumor treatment. Sulfobetaine methacrylate endowed the nanogels with zwitterionic properties in vitro and in vivo, resisting the adsorption of nonspecific proteins, good hemocompatibility, and biocompatibility. Further, NIR laser irradiation accelerated the DOX release rate. Indeed, NIR irradiation of intratumorally injected nanovectors (1–50 mg/mL) achieved a significant increase of antitumor efficiency in H22-bearing mice thanks to a combined effect of chemotherapy and hyperthermia.

Ma et al. [43] realized ternary NPs composed of dendritic carbon dots coated with poly zwitterionic [poly(carboxybetaine methacrylate) for tumor-specific drug delivery. These NPs achieved “stealth” delivery in blood during their transportation toward/inside tumor cells: The extracellular pH induced the conversion of zwitterionic carrier surface to the positive charge, favoring the interaction with the negatively charged cancer cell membrane and enhancing cellular uptake. Following endocytosis, the acidic tumor microenvironment led to fast DOX release. Moreover, the pharmacokinetic studies highlighted a longed half-life in the bloodstream (8–69 h) that promoted drug accumulation into the tumour site. Zwitterionic carboxybetaine was used also to develop functionalized dendrimer-entrapped gold NPs (Au DENPs) for gene delivery [44]. Zwitterionic modification of Au DENPs resisted serum protein. Hence, the vectors retained their small hydrodynamic size and positive charge in serum. Moreover, further modification of CBAA with morpholine (Mor) maintained the antifouling properties and gave lysosome targeting ability, enabling it to inhibit cancer cell metastasis in vitro of HeLa cells at different times 2,4,8 and 12 h.

Another recent example is given by Ding et al. [45] that synthesized pH-responsive polymer NPs coated with poly(carboxybetaine methacrylate) (PCBMA) as antifouling shell and functionalized with RGD to target human glioblastoma (U87) cells. PCBMA conferred low-fouling property and significantly resisted aspecific interaction with HeLa and RAW 264.7 cells. These properties were comparable to the same NPs with PEG, suggesting that PCBMA could represent an advantageous alternative to PEG for antifouling coatings due to the presence of several carboxyl groups available for further surface modifications.

Au NPs was also used as a structural core to anchor zwitterionic triblock polymers in other two studies. The former aimed to design an antifouling pH-responsive nanocarrier [46]. In detail, on the Au core was anchored a triblock copolymer consisting of (i) poly(oligo(ethylene glycol)methyl-ether-methacrylate) POEGMA that favored the formation of a dense inner shell, (ii) poly(2-(diisopropyl-amino)ethyl-methacrylate) PDPA that served as a pH-responsive sponge for encapsulation and release of hydrophobic DOX, and (iii) poly(2-(methacryloyloxy)ethyl phosphorylcholine) PMPC that possessed antifouling features. These nanocarriers greatly exhibited colloidal stability, biocompatibility, and antifouling capability in bio-media. Moreover, the proposed nanocarriers showed enhanced antitumor efficacy at 24 h on the MCF7 breast cancer cell line compared to the free drug. In the latter study [47], AuNPs were covered with three multifunctional building blocks (Tat-R-EK) to develop a tumor microenvironment responsive nanocarrier for cancer radiotherapy. The three-block polymer constituted (i) a zwitterionic peptide sequence (alternative glutamic acid and lysine), (ii) a cell-penetrating and nuclear targeting peptide sequence consisting of Tat peptide (GRKKRRQRRRPQ), and (iii) a peptide sequence (GFLG) that is cleaved by tumor overexpressed cathepsin B. The obtained AuNPs exhibited colloidal stability and negligible immunogenicity and toxicity. Moreover, functionalized NPs responded to tumour overexpressed cathepsin B, with following specific enhancement of tumor cell uptake and following DNA damage upon X-ray irradiation. Another two examples of antifouling pH dual-sensitive AuNPs were proposed for photodynamic treatment of cancer by Wu et al. [48,49]. Additionally, in this case, a zwitterionic peptide sequence of alternate glutamic acid and lysine was used to stealth AuNPs and the prodrug 5-aminolevulinic acid (ALA) anchored on its surface. The zwitterionic peptide coating ensured colloidal stability and low specific protein adsorption to AuNPs in a complex physiological environment. Moreover, the drug release from the nanocarriers was not observed in blood circulation but was induced after cell internalization by acidic lysosomal compartment, with successful photodynamic ablation of cancer cells compared to free ALA. Recently Li et al. [50] proposed dendrimer-entrapped Au NPs as a multifunctional platform to trigger a dual anti-inflammatory and antioxidative response for arthritis rheumatoid treatment. In detail, G5 PAMAM dendrimers were conjugated with PEGylated FA (PEG-FA) for targeting specificity, modified with 1, 3-propane sultone (1,3-PS) for antifouling properties, and loaded with alpha-tocopheryl succinate (PEG-αTOS) and tumor necrosis factor-α (TNF-α) siRNA, for its antioxidative and anti-inflammatory activity, respectively. The proposed dendrimer showed cytocompatibility and inhibited TNF-α expression in macrophages in vitro. In addition, when tested in vivo, the treatment achieved promising therapeutic effects in inflammatory cytokines downregulation of rheumatoid arthritis lesions. Moreover, the Au concentration in joints (164.40 μg/g) significantly decreased at four days after injection while gradually increasing in the liver. Although exhibiting a rapid clearance, the multifunctional dendrimers showed a retention time sufficiently long to carry out their therapeutic effect.

Liu et al. [51] developed Carbon-Quantum-Dots-loaded Mesoporous Silica NPs equipped with a zwitterionic antifouling layer made of oppositely charged functional groups (COO^−^ and –HN^+^(Me)_2_). Besides, to achieve an enzyme-controlled cargo release, the MSN pores were filled with esterase degradable, polycaprolactone (PCL), and DOX. The anionic part of the zwitterionic layer was designed to switch to positive charge at tumoral acidic environmental (pH value < 6.8). The antifouling layer reduced nonspecific protein absorption to 1/10 compared to unmodified NPs, while it facilitated the tumor cellular uptake. Moreover, at tumor acidity, the release of the DOX dramatically increased over time, reaching over 90% in three days, indicating high selective drug delivery performance of nanocarriers to tumor tissue.

Chen et al. [52] designed a polymer by combining zwitterionic molecule poly(2-methylacryloyloxyethyl phosphorylcholine) (pMPC), with a membrane-inspired structure, loaded with a chemotherapeutic prodrug poly(10-hydroxy-camptothecin methacrylate) (pHCPT) that possesses a broad spectrum of antitumor activity. The amphiphilic polymer pMPC-b-pHCPT self-assembled into micelles. The hydrophilic outer shell prevented the absorption of albumin and other molecules. The length of poly-prodrug block determined the cellular internalization of micelles. Moreover, the hydrophilic layer prevented rapid clearance and prolonged blood retention time in vivo (47.0% and 35.8% for 24 h), promoting the accumulation of proposed micelles in tumor sites through EPR with better chemotherapeutic efficacy (reduction of tumor volume around 27.7 ± 26.4%) than the free drug. Besides, pMPC was also used to modify the surface of stimuli-responsive core-shell NPs made of gelatin and carrying DOX [53]. The pMPC shell prevented non-specific protein adsorption shielding the positive charged of the inner core. Under acidic conditions of the tumor microenvironment, the zwitterionic coating was hydrolyzed following the disclosing of positive charge, that favored uptake (∼7.2% within 1 h) and enhanced anticancer therapeutic efficacy of nanocarrier. Zwitterionic Janus Dendrimers were proposed for therapeutic protein delivery in vivo [54]. Zwitterionic dendrons exposed on the surface avoided serum protein adhesion in the biological environment. Then, Zwitterionic Janus Dendrimers containing biomimicking glyceryl phosphoryl choline (GPC) showed favored biocompatibility, enhanced cancer-specific cell uptake, efficient lysosomal escape, and longer pharmacokinetic profiles compared to the PEGylated nanocarriers.

#### 3.3.3. Other Strategies

Chen et al. [55] designed crystallized iron oxide NPs (HCIONPs) coated with a polysiloxane-containing diblock copolymer (PEO-b-PγMPS) for photothermal therapy. Polymer coating conferred colloidal stability under various physiological conditions and improved evasion of the immune system increasing the accumulation of magnetic NPs into tumor tissue after injection of 20 mg/kg of Fe.

Shi et al. [56] developed antifouling silver NPs (AgNPs) to modulate the immune response in diabetic wound healing in vitro and in vivo. Here, the authors obtained, mixing chitosan and dextran with AgNPs, an antifouling coating able to improve bactericidal properties of AgNPs. This hydrogel prevented adhesion to the wound, pushed immune cells into wound regions, and provided a sustained release of Ag^+^. Recently, Huang et al. [57] designed amphoteric natural starch as a shell for coating polymeric NPs. Even if starch is usually negatively charged, here it was etherified to bring both anionic and cationic groups with a net positive charge. The starch-coated NPs showed high targeting, PC-free properties even at high levels of serum medium, and good cell internalization capabilities after 0.1 mg/mL of treatment. Moreover, NPs were stable over six months of storage and maintained their antifouling properties at 37 °C (human body temperature), under mimicking dynamic flow conditions. The starch-coated NPs functionalized with anti-CD44 showed targeting efficiency and high cell internalization demonstrating their potential use as photodynamic therapy drug carriers.

## 4. Discussion

It is now accepted that the tailoring of morphological characteristics of NPs such as shape, size, and surface improve their performance for diagnostic and/or therapeutic application. In a biological context, NPs adsorb, in a non-specific manner, various proteins such as apolipoproteins, immunoglobulins, and complement proteins on the surface as a consequence of the opsonization [58,59]. This process, known as PC, controls the fate of NPs in vivo because it represents the interface with cells and influences their physiological response such as cellular absorption, inflammation, accumulation, degradation, and targeting efficiency [60,61]. Furthermore, after unspecific protein adhesion, the NPs are recognized by the mononuclear phagocytic system with a substantial decrease in circulation times in blood, hampering their in vivo performances [62,63]. Hence, the research of novel biocompatible nanomaterials to synthesize antifouling NPs is one of the current goals. Antifouling coatings aim to avoid protein adhesion and consequently improve their therapeutic and diagnostic capability for in vivo purposes favoring the formation of a biolayer of water molecules (H_2_O) on their surface.

Here, we systematically analyzed the state of the art of stealth strategies developed to date, able to block the undesirable adsorption of biological macromolecules to achieve a reliable application of NPs for diagnosis and/or therapy. Our systematic review groups all studies in which antifouling strategies were assessed in vitro and/or in vivo as diagnostic or therapeutic tools. Starting from 2009 to date, we identified 122 records. Among them, we selected for our qualitative study 43 studies meeting the inclusion criteria that we set. Thus, we grouped the eligible studies according to the main application: 22 studies fall in antifouling strategies for diagnostic purpose, and the other 21 studies regard antifouling strategies for therapeutic purpose. Within these two macro-areas, we subdivided the works according to the antifouling approach used pegylated, zwitterionic, or others. PEGylation is one of the most common procedures to minimize opsonization. PEGs, binding to large amounts of H_2_O molecules, act as barriers hampering protein adsorption on the surface. Although the PEGylation strategy of NPs improves the stability in biological samples, it is not completely able to avoid the adsorption of biomolecules [64,65,66]. Therefore, although functionalization with PEG is traditionally one of the most common procedures to minimize opsonization, nowadays it appears less investigated. From our systematic analysis of literature emerges that only a few studies choose PEGylation strategy (20%), while about 62% of the selected studies adopted zwitterionic strategy to endow the NPs of antifouling properties. Moreover, studies that investigate PEGylation strategy are also older ones. In the examined papers, antifouling properties of PEG resulted in being more exploited for imaging purposes, PEG has been associated with targeting ligands [37], and has been conjugated with other kinds of antifouling covers [19,38], with different charged terminal groups [24,25], or with photoresponsive polymeric shell [39]. However, high salt conditions induce aggregation of PEGylated [67], and the PEG polymers, increasing the hydrodynamic diameter of NPs, may reduce the access of NPs to confined spaces and also prevent renal clearance in vivo [68]. Furthermore, the inability of simple PEG layers to completely avoid protein adsorption has pushed towards new and alternative antifouling approaches. Among all systematically reviewed antifouling strategies, zwitterions emerged as those increasingly popular, adopted in 53% of the studies examined for diagnosis and even 70% for therapeutic purposes. Zwitterions are polyelectrolytes that contain both positive and negative counterparts on the same chain, but with an overall neutral charge. Through strong ionic structuring of water, they prevent protein absorption [69]. Furthermore, the presence of different functional groups allows broadening the range of applications in the diagnostic and therapeutic field, developing multimodal [28] and theranostic agents [22]. The zwitterions typically have a quaternary ammonium cation, while based on the type of sulfonate, carboxylate, or phosphate anion differentiate into sulfobetaine (SB) [42], carbossibetaine (CB) [43], and fosforilcoline (PC) [53]. Other natural zwitterions are also aminoacids in the right pH range [41]. Antifouling zwitterionic coatings improved biocompatibility and bioavailability of metallic NPs [22], polymeric micelles [52], or dendrimers [27] and showed marked stability in vitro and/or in vivo over a high salt concentration and broad range of pHs. Furthermore, the antifouling zwitterionic strategy not only leads to low protein absorption (typically assessed in vitro by using FBS) [44] and non-specific cellular adhesion, but also it reduces RES uptake [52,54]. Furthermore, the half-lives and clearance for zwitterionic-coated NPs (sub-10 nm) have been found to be relatively short from minutes to hours [47]. Although zwitterions molecules showed non-specific interactions with macrophages, they do not recognize a specific protein on membrane cells, with this being one reason that a number of authors have set active targeting conjugating zwitterions with specific moieties (i.e., whole IgG antibodies) [31]. By using a short link chain for the second functional group, non-fouling properties of zwitterionic NPs are maintained. Several authors have also investigated the pH-responsiveness of zwitterionic NPs slightly acidic pH to control the release of therapeutics. The protonation of a tertiary amine group showed a charge reversal property of zwitterionic NPs improving cell association in a tumor microenvironment (pH 6.8) [45,48,49,53]. Finally, some studies have investigated not zwitterionic coatings with positively and negatively charged ligands (1:1 ratio) that are able to block protein adhesion. For example, the use of cationic chitosan and anionic dextran (CNDM) reached an antifouling property [56].

## 5. Conclusions

To conclude, the surface of NPs can be finely engineered with antifouling coatings to tune their pharmacokinetic properties in accordance with their biomedical applications. We have systematically reviewed the strategies developed to improve NPs antifouling behavior, and between these, PEGylation and zwitterionic polymers resulted in being the most assessed macro areas. It is not easy to compare PEG and zwitterionic coatings and define what is better for clinical practice because several factors (such as thickness, charge, length, or grafting density), and not just one, affect their whole antifouling behavior and the following interaction with biological systems.

Nevertheless, from our systematic revision, it appears that PEGylation strategy is less used and it mostly exploited for the development of nanosized diagnostic tools rather than therapeutic ones. Although PEG has been the most widespread strategy for a long time, due to its ease of handling and low costs, it suffers some limitation compared to zwitterionic approach as it can involve aggregation and, above 35 °C, does not always succeed incompletely avoiding the formation of PC. Moreover, the major hurdle consists in the immune response caused by antibodies formation against PEG, which can hinder the efficiency of the PEGylated nanovectors. On the other hand, we showed the recent widespread trends of the application of stimuli-responsive zwitterionic vectors, that have enhanced drug delivery with limited side effects. Moreover, zwitterionic ligands showed good biocompatibility and are easily functionalized.

Nevertheless, in vivo biofouling is a complex dynamic process with many aspects that need yet to be explored for increasing clinical applications of NPs. Future issues should be addressed to improve a coating design to obtain zwitterionic nanovectors with different functions without altering antifouling properties. Furthermore, processing costs should be reduced, and the synthesis process simplified in order to have larger scale-up.

However, despite the growing potential utility of NPs in diagnostics and therapeutic strategies, and the growing number of successful examples of biocompatible architectures, NPs face biological and technological limitations that must be addressed to achieve consistent clinical impact. This gap is intrinsically related to their small size, that, if on one hand, confers great potentials and promises, on the other one hand entails critical scientific challenges for their clinical translation.

## Figures and Tables

**Table 1:** Characteristics of included studies on antifouling nanoparticles (NPs) in diagnostic.

Author	Year	Nanostructure	Antifouling Moiety	In Vitro Model	In Vivo Model	Application
Cho et al.	2009 [15]	Iron Oxide NPs (TCL-SPION)	poly(3-(trimethoxysilyl)propyl methacrylate-r-PEG methylethermethacrylate-r-N-acryloxysuccinimide)	-	BALB/c mice	Optical imaging (OI)
Oh et al.	2011 [16]	Tantalum Oxide (TaOx NPs)	Polyethylen Glycol (PEG)	RAW264.7	Rats	Bimodal imaging (Computed Tomography (CT) and OI)
Liu et al.	2014 [17]	Hybrid Lutetium Oxide NPs (UCNPs)	PEG	MCF-7	Kunming mice, C57BL/6 mice, and wister rats	Multimodal imaging (up-conversion luminescent, X-ray and Magnetic Resonance Imaging (MRI))
Joeng et al.	2013 [18]	Poly (oxyethylene galactaramide)s (PEGA) NPs	PEGA	Hela	SCC7 tumour-bearing mice	OI
Li et al.	2015 [19]	Magnetic Iron Oxide NPs (IONPs)	PEG and allyl glycidyl ether (PEG-b-AGE)	RAW264.7, D556, Daoy, U87MG, MDA-MB-231, MCF7 and A549	-	Theranostic
Tu et al.	2016 [20]	Silicon Quantum DotNanoparticles (SiQD-NPs)	PEG and Bovine Serum Albumin (BSA)	CHO, SKOV3	-	OI
Suàrez-Garzìa	2021 [21]	Metal-phenolic NPs (MPS)	PEG	CT26, HeLa, 3T3	bearing CT26 tumour-bearing mice	SPECT/PET
Li et al.	2019 [22]	Gold NPs (AuNPs)	L-cysteine-functionalized with poly(but-3-yn-1-yloxy)-2-oxo- 1,3,2-dioxaphospholane (zPBYP)	RAW264.7	-	Theranostic
Sui et al.	2020 [23]	Ultrasmall Gadolinium oxide NPs Gd_2_O_3_ NPs)	PEG-L-cysteine	RAW264.7	B16 lung cancer metastasis mouse model	MRI
Wang et al.	2017 [24]	Manganese Oxide NPs (Mn_3_O_4_NPs)	PEG-L-cysteine	C6 and Raw 264.7	Mouse	MRI
Ma et al.	2017 [25]	Iron Oxide NPs (Fe_3_O_4_NPs)	PEG-L-cysteine	L929	Rats	MRI
Wang et al.	2019 [26]	Mn_3_O_4_ NPs	L-lysine	KB	Mouse	MRI
Xiong et al.	2017 [27]	Dendrimer-entrapped gold NPs (Au DENPs)	Carboxybetaine Acrylamide (CBAA)	U87MG	U87MG tumour-bearing mice	X-ray CT
Liu et al.	2019 [28]	Gadolinium(-Complexed Dendrimer-Entrapped Gold NPs (Gd-Au DEN-PS.)	CBAA, 2-methacryloyloxyethyl phosphorylcholine (MPC) or 1,3-propane sultone (1,3-PS)	Macrophage	B16 lung cancer metastasis mouse model	Bimodal imaging (X-ray CT and MRI)
Zhu et al.	2019 [29]	poly(cyclotriphosphazene-co-polyethylenimine) nanospheres (PNSs)	1,3-PS	4T1 cells	4T1 tumor-bearing mouse	Theranostic
Ferretti et al.	2020 [30]	IONPs	Zwitterionic Dopamine Sulfonate (ZDS)	BV2 and glial cells	CD-1 mice	MRI
Tasso et al.	2015 [31]	Quantum dot (QD)	Poly(methacrylamidosulfobetaine-block-4-vinylimidazole)	HEK293	-	OI
Wang et al.	2014 [32]	Superparamagnetic Iron Oxide NPs (SPIO)	BSA	PDAC, Panc-1 and L02	-	MRI
Lamanna et al.	2011 [33]	IONPs	Phosphonate	U87MG	Rats	Multimodal imaging (OI and MRI)
Cotin et al.	2019 [34]	IONPs	Dendron coating	Huh7	CD-1 mice	Theranostic
Karmali et al.	2012 [35]	SPIO	Crosslinked dextran	-	C57BL/6J mice	MRI
Chen et al.	2013 [36]	IONPs	Poly(ethylene oxide)-block-poly(γ-methacryloxypropyltrimethoxysilane) (PEO-b-PγMPS)	SK-BR-3, MDA-MB-231, MCF-7, MDA-MB-453, 4T1 and RAW264.7	4T1 mice	MRI

**Table 2:** Characteristics of included studies on antifouling NPs in therapy.

Author	Year	Nanostructure	Antifouling Moiety	In Vitro Model	In Vivo Model	Application
Lv et al.	2017 [37]	Lipidic NPs	polyethylene glycol (PEG)	SKOV-3	-	Targeted drug delivery
Park et al.	2015 [38]	poly(ethyleneimine) (aPEI) NPs	PEG	Hela	SCC7 tumor bearing mice	Drug delivery
Yao et al.	2017 [39]	Poly(D,L-lactide-co-glycolide) (PLGA) NPs	PEG-hydrophilic block, hexadecyl hydrophobic block, and a 2-nitrobenzyl linker	HepG2 and HeLa	HepG2 tumour-bearing nude Balb/c mice	Drug delivery
Elsabahy et al.	2013 [40]	Polyphosphoester (PPE) micelle	Zwitterionic diblock copolymers (acrylic acid/amino group (1:1))	RAW 264.7	-	Drug delivery
Huang et al.	2016 [41]	Hollow gold-silver nanoshells	Cysteine betaine (Cys-b)	MDA-MB-453	-	Hyperthermia
Zheng et al.	2020 [42]	Poly(N-isopropylacrylamide) (PNIPAM) Nanogels	Sulfobetaine methacrylate (SBMA)	L929 and HepG2	H22-bearing mice	Photothermal drug delivery
Ma et al.	2018 [43]	Dendritic carbon dots (CDs)	Poly(carboxybetaine methacrylate) (pCBMA)	4T1 and HepG2	BALB/c mice	Drug delivery
Xiong et al.	2019 [44]	Dendrimer-entrapped gold NPs (Au DENPs)	Carboxybetaine acrylamide (CBAA)	Hela	-	Gene delivery
Ding et al.	2019 [45]	poly(2-(diisopropylamino)ethyl methacrylate) (PDPA)NPs	pCBMA	RAW 264.7, HeLa, and U87	-	Targeted drug delivery
Ellis et al.	2017 [46]	Gold NPs (Au NPs)	Poly(2-(methacryloyloxy)ethyl phosphorylcholine) pMPC	MCF-7	-	Drug delivery
Ding et al.	2020 [47]	Au NPs	Peptide sequence of glutamic acid and lysine	LM3	LM3 Tumour-bearing mice	Radiotherapy
Wu et al.	2017 [48]	Au NPs	Peptide sequence of glutamic acid and lysine	SCC-7	SCC-7 tumour-bearing mice	Photodynamic therapy
Wu et al.	2017 [49]	Au NPs	Peptide sequence of glutamic acid and lysine and RGD moieties	A549	-	Photodynamic therapy
Li et al.	2020 [50]	Au DENPs	1,3-propane sultone (1,3-PS)	RAW264.7	Collagen-induced arthritis (CIA) mouse	Targeted drug delivery
Liu et al.	2016 [51]	Mesoporous Silica NPs	-COO^−^ and -HN^+^(Me)_2_	SCC, HaCaT	-	Theranostic
Chen et al.	2017 [52]	poly(10-hydroxy-camptothecin methacrylate (pMPC-b-pHCPT) Micelles	pMPC	HeLa and L929	Kunming mice and nude mice	Drug delivery
Chen et al.	2019 [53]	Cationic gelatin NPs ((+)GNPs)	pMPC	HeLa	SD rats and nude mice	Targeted drug delivery
Wang et al.	2019 [54]	Janus dendrimer (JD GPC)	Glycerylphosphorylcholine (GPC)	HT-29, SKOV-3, U87	BALB/c mice,	Drug delivery
Chen et al.	2014 [55]	Crystallized iron NPs (HCIONPs)	Poly(ethylene oxide)-block-poly(γ-methacryloxypropyltrimethoxysilane) (PEO-b-PγMPS)	SUM-159	SUM-159 tumor-bearing BALB/c mice	Targeted drug delivery
Shi et al.	2018 [56]	Silver NPs (Ag NPs)	Chitosan and dextran	NIH 3T3	SD rats	Targeted drug therapy
Huang et al.	2020 [57]	(Etherified starch-coated poly(methyl methacrylate- co-acrylic acid) Micelles	Amphoteric starch	SW480 and A549	-	Targeted drug therapy

## Supplementary Materials

The following are available online at https://www.mdpi.com/2079-4991/11/3/780/s1, S1. Key terms used in literature search; S2. PRISMA (Preferred Reporting Items for Systematic Reviews and Me-ta-Analyses) Checklist.; Table S1. PRISMA Checklist for Preferred Reporting Items for Systematic Reviews and Meta-Analyses.
